# The Histone Deacetylases Hst1 and Rpd3 Integrate De Novo NAD^+^ Metabolism with Phosphate Sensing in *Saccharomyces cerevisiae*

**DOI:** 10.3390/ijms24098047

**Published:** 2023-04-28

**Authors:** Benjamin Groth, Yi-Ching Lee, Chi-Chun Huang, Matilda McDaniel, Katie Huang, Lan-Hsuan Lee, Su-Ju Lin

**Affiliations:** Department of Microbiology and Molecular Genetics, College of Biological Sciences, University of California, Davis, CA 95616, USA; bgroth@ucdavis.edu (B.G.); ycclee@ucdavis.edu (Y.-C.L.); anchuang@ucdavis.edu (C.-C.H.); mmcdaniel@ucdavis.edu (M.M.); kahua@ucdavis.edu (K.H.); lhslee@ucdavis.edu (L.-H.L.)

**Keywords:** histone deacetylase, NAD^+^ metabolism, yeast genetics, gene regulation

## Abstract

Nicotinamide adenine dinucleotide (NAD^+^) is a critical cofactor essential for various cellular processes. Abnormalities in NAD^+^ metabolism have also been associated with a number of metabolic disorders. The regulation and interconnection of NAD^+^ metabolic pathways are not yet completely understood. By employing an NAD^+^ intermediate-specific genetic system established in the model organism *S. cerevisiae*, we show that histone deacetylases (HDACs) Hst1 and Rpd3 link the regulation of the de novo NAD^+^ metabolism-mediating *BNA* genes with certain aspects of the phosphate (Pi)-sensing *PHO* pathway. Our genetic and gene expression studies suggest that the Bas1–Pho2 and Pho2–Pho4 transcription activator complexes play a role in this co-regulation. Our results suggest a model in which competition for Pho2 usage between the *BNA*-activating Bas1–Pho2 complex and the *PHO*-activating Pho2–Pho4 complex helps balance de novo activity with *PHO* activity in response to NAD^+^ or phosphate depletion. Interestingly, both the Bas1–Pho2 and Pho2–Pho4 complexes appear to also regulate the expression of the salvage-mediating *PNC1* gene negatively. These results suggest a mechanism for the inverse regulation between the NAD^+^ salvage pathways and the de novo pathway observed in our genetic models. Our findings help provide a molecular basis for the complex interplay of two different aspects of cellular metabolism.

## 1. Introduction

Nicotinamide adenine dinucleotide (NAD^+^) is an essential enzymatic cofactor. NAD^+^, its reduced form NADH, and the phosphorylated derivative NADP^+^ serve a wide variety of critical roles in the cell. NAD^+^/H is an oxidative electron acceptor in central metabolism, a source of electrons for mitochondrial respiration [1,2], and a cofactor for the sirtuin class of histone deacetylases [3,4,5] and for the poly-ADP-ribose polymerase (PARP) class of DNA repair enzymes [6]. Owing to its centrality and far-ranging influence in the cell, perturbations to NAD^+^ homeostasis are associated with a considerable and diverse number of diseases, including various metabolic disorders [7], neurological disorders [8], cardiovascular disease [9], and numerous cancers [10,11,12]. Supplementation of NAD^+^ precursors has been shown to be efficacious in treating or alleviating symptoms of several diseases [13,14,15].

Owing to the complex regulatory networks governing the assimilation of these compounds into the cell’s NAD^+^ pool however, treatment of many disorders involving defective NAD^+^ homeostasis is not always straightforward. In addition, NAD^+^ precursors may interact with a large variety of signaling pathways in the cell, including diverse types of nutrient sensing [16,17,18,19] as well as inflammation and various other responses to infection [20]. As such, it is vital to understand the regulation of NAD^+^ metabolism and its interaction with other cellular processes.

**Figure 1:**
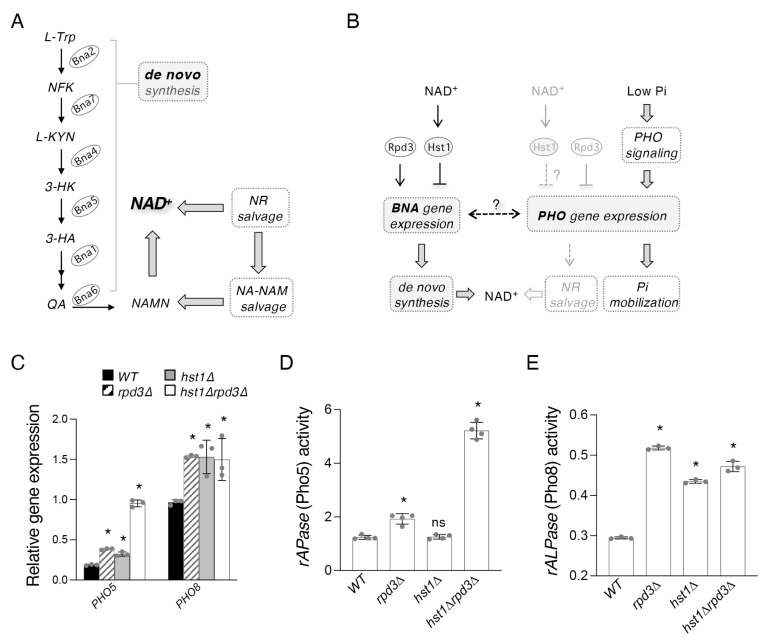
Rpd3 and Hst1 negatively regulate the *PHO* pathway genes *PHO5* and *PHO8*. (**A**) Abridged model of the NAD^+^ biosynthetic pathways in *Saccharomyces cerevisiae*. De novo NAD^+^ metabolism begins with TRP, which is converted into NaMN by the Bna enzymes (Bna2, -7, -4, -5, -1, -6) (left). NaMN is also produced by salvage of NA and NAM, which is further connected with salvage of NR (right). NA, nicotinic acid; NAM, nicotinamide; NR, nicotinamide riboside; QA, quinolinic acid; TRP, L-tryptophan; NFK, N-formylkynurenine; KYN, kynurenine; 3-HK, 3-hydroxykynurenine; 3-HA, 3-hydroxyanthranilic acid; ACMS, 2-amino-3-carboximuconate-6-semialdehyde. KA, kynurenic acid; NaMN, nicotinic acid mononucleotide. Abbreviations of protein names are shown in ovals. Bna2, tryptophan 2,3-dioxygenase; Bna7, kynurenine formamidase; Bna4, kynurenine 3-monooxygenase; Bna5, kynureninase; Bna1, 3-hydroxyanthranilate 3,4-dioxygenase; Bna6, quinolinic acid phosphoribosyl transferase. (**B**) Relationship between de novo NAD^+^ biosynthesis, NR salvage, and Pi sensing. Rpd3 and Hst1 antagonistically regulate the *BNA* genes of the de novo pathway and have previously been shown to negatively regulate certain *PHO* targets including *PHO5* and *PHO8* phosphatases. *PHO* gene expression is sensitive to cellular phosphate (Pi) levels and is induced by Pi-depletion. Two *PHO* targets, the acid phosphatase Pho5 and the alkaline phosphatase Pho8, are known to convert NMN to NR, producing Pi. *PHO* signaling is mediated by the transcription factor complex formed by Pho2 and Pho4. (**C**) Gene expression qPCR analysis of *PHO5* and *PHO8* levels in WT, *rpd3*Δ, *hst1*Δ, and *hst1*Δ*rpd3*Δ cells. Values shown are relative expression levels normalized to *TAF10* as a control. *PHO5* expression is increased in *rpd3*Δ and *hst1*Δ cells and is especially strongly raised in *hst1*Δ*rpd3*Δ cells. *PHO8* expression in *rpd3*Δ, *hst1*Δ, and *hst1*Δ*rpd3*Δ cells is raised to a similar extent relative to WT cells. (**D**) Pho5 (repressible acid phosphatase, *rAPase*) activity in WT, *rpd3*Δ, *hst1*Δ, and *hst1*Δ*rpd3*Δ cells. Both *hst1*Δ and *rpd3*Δ cells show a significant increase in Pho5 activity relative to WT. The *hst1*Δ*rpd3*Δ double mutants show a further increase of Pho5 activity relative to the single mutants. (**E**) Pho8 (repressible alkaline phosphatase *rALPase*) activity in WT, *rpd3*Δ, *hst1*Δ, and *hst1*Δ*rpd3*Δ cells. *rpd3*Δ, *hst1*Δ, and *hst1*Δ*rpd3*Δ cells show comparable increases in Pho8 activity relative to WT cells. For (**C**–**E**), the graphs are representative of the trend observed across three independent experiments. Error bars represent data from three technical replicates for each strain in an experiment. The *p* values are calculated using Student’s *t*-test (*, *p* < 0.05; ns, not significant).

Biosynthesis of NAD^+^ in budding yeast proceeds through the following pathways: NA-NAM (nicotinic acid-nicotinamide) salvage, NR (nicotinamide riboside) salvage, and de novo biosynthesis of QA (quinolinic acid) from L-tryptophan [21]. In the first pathway, NAM is deaminated to NA by Pnc1 [22], followed by phosphoribosylation to NaMN (nicotinic acid mononucleotide) by Npt1 [5], adenylation to NaAD (deamindo-NAD^+^) by Nma1/Nma2 [23,24], and, finally, amination to NAD^+^ by Qns1 [25]. NR salvage may merge with NA-NAM salvage via conversion to NAM by the nucleosidases Urh1 and Pnp1, or by phosphorylation to NMN by Nrk1 [26,27], followed by adenylation to NAD^+^ by Pof1 and Nma1/Nma2 [28]. De novo metabolism is mediated by the *BNA* genes and results in the formation of NaMN, at which point the pathway merges with NA/NAM salvage (Figure 1A).

De novo metabolism, in addition to contributing toward the cell’s NAD^+^ pool, has a complex and reciprocal relationship with other signaling networks in the cell. The flux of specific de novo metabolites, such as kynurenine (L-KYN) and 3-hydroxykynurenine (3-HK), is often affected, and itself in turn affects various signaling events induced by infection, inflammation, and nutrient sensing [20]. Budding yeast represents a relatively simpler model in which to investigate the relationship of de novo activity with other aspects of NAD^+^ biosynthesis and other cellular processes in general. The possibility of cross-talks between de novo metabolism and other branches of NAD^+^ biosynthesis may be a promising avenue of investigation. The NAD^+^-dependent sirtuin histone deacetylase Hst1, for instance, is itself a regulator of de novo NAD^+^ biosynthesis [19,29,30], linking de novo activity with the status of the cell’s NAD^+^ pool.

Although detailed mechanisms are not completely understood, several nutrient-sensing pathways have been connected with the regulation of NAD^+^ homeostasis. For example, NR salvage activity is associated with the activation of the phosphate (Pi)-sensing *PHO* pathway [16]. In response to Pi-depletion, the Pho2–Pho4 transcription complex activates *PHO* target genes [31,32] (Figure 1B), which leads to increased production of NR [16]. Interestingly, *PHO* activation appears to be inhibited by NaMN accumulation because mutants defective in NaMN production and NA-NAM salvage, such as the *npt1*Δ and *pnc1*Δ mutants, show increased *PHO* activity [16]. However, it remains unclear whether altered NAD^+^ levels and/or specific NAD^+^ intermediates have a more direct role in regulating *PHO* activity. *PNC1* has been shown to be a target of regulation by the cAMP-PKA pathway through activation mediated by the Msn2/4 transcription factors [21]. It has also been suggested that *PNC1* expression may decline in response to *PHO* activation through a pathway mediated by stress-response transcription factors Rim15 and Msn2/4 [21]. However, direct evidence supporting the role of Pnc1 in connecting *PHO* and NAD^+^ metabolism has not been shown. Moreover, a multifaceted relation between de novo NAD^+^ metabolism, de novo adenine biosynthesis (*ADE*), and NA-NAM salvage has been reported in adenine-depleted cells and cells harboring mutations mimicking adenine depletion such as the *ade16*Δ*ade17*Δ mutants [18]. *PHO* activation has also been reported in *ade16*Δ*ade17*Δ cells [33]. In these cells, activation of *BNA* and *ADE* genes are co-regulated by the Bas1–Pho2 transcription complex [18], while activation of *PHO* genes is mediated by the Pho2–Pho4 complex [33]. It is suggested that Pho2 may help coordinate the regulation of *ADE* and *PHO* genes because Pho2 is shared between the Bas1–Pho2 (for *ADE*) and Pho2–Pho4 (for *PHO*) complexes. Since most of these studies were carried out under adenine-depleted conditions and/or in the *ade16*Δ*ade17*Δ mutants, the role of Pho2 in coordinating these pathways in wild-type cells under normal conditions and whether additional factors are involved remains unclear.

In this study, we aim to study how de novo NAD^+^ metabolism is connected to *PHO* signaling with a particular focus on Pho2, *PNC1*, and two histone deacetylases (HDACs), Hst1 (NAD^+^-dependent) and Rpd3. In previous work, we identified the histone deacetylase Rpd3 as a positive regulator of de novo NAD^+^ metabolism, which acts specifically by antagonizing the Hst1-dependent repression of the *BNA* genes (Figure 1B) [30]. Rpd3 and Hst1 also appear to co-regulate many other additional NAD^+^ metabolic factors either antagonistically or synergistically [30], which suggests these two HDACs might coordinate de novo NAD^+^ metabolism with other branches of the NAD^+^ metabolic network or with other signaling pathways in the cell. Our previous study showed that the expression of several *PHO* genes is increased in *hst1*Δ mutant cells [19], suggesting a direct role of Hst1 in the repression of *PHO* genes in an NAD^+^-dependent manner. Rpd3 appears to also repress the *PHO* pathway [34,35,36], which is opposite to its role as a positive regulator for *BNA* gene expression [30] (Figure 1B). The interaction of Hst1 and Rpd3 on the regulation of *PHO* genes is currently unclear. Here, we present evidence that Hst1, Rpd3, and the transcription factor Pho2 link the regulation of the *BNA* genes with *PHO* signaling and with NR and NA-NAM salvage, in which a novel role for Pho2 as a regulator of *PNC1* is also identified. This work contributes to the elucidation of NAD^+^ metabolism, its regulation, and its relationship with other metabolic pathways in the cell.

## 2. Results

### 2.1. Hst1 and Rpd3 Regulate Targets of the PHO Pathway

To further understand the interaction of these two HDACs on the regulation of *PHO* genes, we first determined the expression of the *PHO5* and *PHO8* genes (encoding two *PHO*-regulated phosphatases) in cells lacking either one or both HDACs. *PHO5* and *PHO8* are chosen because they have been shown to be able to convert NMN to NR, and therefore contribute to NR salvage [16,37]. Interestingly, we found that levels of *PHO5* expression in *hst1*Δ*rpd3*Δ cells are strikingly increased compared to WT, *rpd3*Δ, and *hst1*Δ cells, suggesting novel synergistic regulation of *PHO5* by Hst1 and Rpd3 (Figure 1C). Moreover, this large increase of *PHO5* expression in *hst1*Δ*rpd3*Δ cells closely resembles the similarly elevated production of NR (a product of Pho5 activity) previously observed in this strain [30]. On the other hand, although *PHO8* expression was also increased in *rpd3*Δ and *hst1*Δ cells, it was not further increased in the double mutant (Figure 1C). Notably, *rpd3*Δ cells show increased *PHO8* expression despite previous work demonstrating that Rpd3 does not affect the acetylation status of the *PHO8* promoter [38], newly establishing a potential role for Rpd3 as a regulator of *PHO8* and suggesting an atypical form of regulation by Rpd3 at the *PHO8* promoter. We next directly determined the phosphatase activities of Pho5 (Figure 1D) and Pho8 (Figure 1E), which also supported the expression results. These results suggest that Rpd3 and Hst1 are regulators shared between the de novo *BNA* genes and the *PHO* genes, which employ markedly different regulatory strategies across contexts.

### 2.2. The Bas1–Pho2 Complex Plays a Role in Hst1- and Rpd3-Mediated Regulation of De Novo NAD^+^ Metabolism

**Figure 2:**
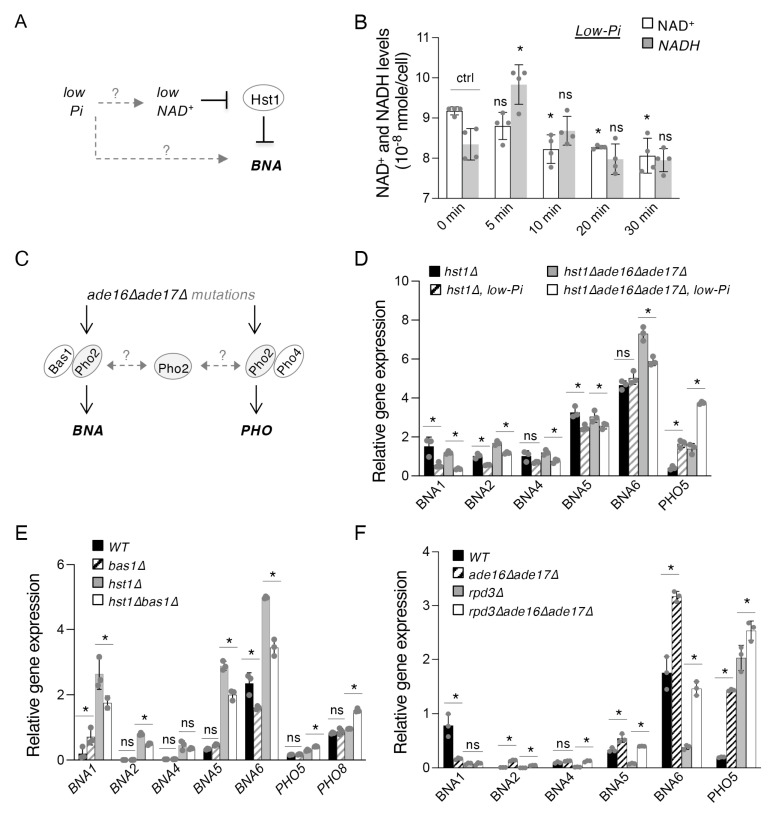
Rpd3 and Hst1 interact with cellular phosphate sensing and the Bas1–Pho2 complex during the regulation of de novo NAD^+^ metabolism. (**A**) Model of the proposed relationship between Pi depletion and reduction of cellular NAD^+^ pools. Pi depletion consequently leads to lower NAD^+^ levels. The limitation of NAD^+^ is predicted to reduce the activity of Hst1, which is an NAD^+^-dependent HDAC. Loss of Hst1 activity would then lead to decreased silencing of the *BNA* promoters. It is unknown whether Pi depletion affects *BNA* expression independent of Hst1. (**B**) NAD^+^ and NADH levels of WT cells grown in low Pi medium over the course of 30 min. Cells transferred into SC medium without Pi rapidly begin to show altered NAD^+^/NADH homeostasis. (**C**) Model depicting the interconnection of the Bas1, Pho2, and Pho4 transcription factors. Pho2 is shared between the *BNA*-activating Bas1–Pho2 complex and the *PHO*-activating Pho2–Pho4 complex. The formation of both complexes is promoted by the adenine precursor ZMP, which is accumulated in *ade16*Δ*ade17*Δ cells. (**D**) Pi depletion reduces the expression of the *BNA* genes in *hst1*Δ and *hst1*Δ*ade16*Δ*ade17*Δ backgrounds. Cells transferred to low-Pi medium for 30 min show reduced expression of several *BNA* genes relative to cells maintained in standard SC medium. *PHO5* is included as a positive control for *PHO* activation. Values shown are relative expression levels normalized to *TAF10* as a control. (**E**) Deletion of *BAS1* reduces expression of the *BNA* genes but increases *PHO5* and *PHO8* expression in an *hst1*Δ background. Deletion of *BAS1* does not significantly affect *BNA* expression in WT cells. (**F**) Deletions of *ADE16* and *ADE17* increase the expression of most *BNA* genes in WT and *rpd3*Δ cells. Rpd3 is required for optimal *BNA* expression in both WT and *ade16*Δ*ade17*Δ cells. *PHO5* is included as a positive control for *PHO* activation. For (**B**,**D**–**F**), the graphs are representative of the trend observed across three independent experiments. Error bars represent data from two biological replicates each with two technical replicates (for (**B**)) or from three technical replicates (for (**D**–**F**)) for each strain in an experiment. The *p* values are calculated using Student’s *t*-test (*, *p* < 0.05; ns, not significant; ctrl, control).

To further understand the mechanisms of the interconnections between de novo NAD^+^ metabolism and the *PHO* pathway, we first sought to determine the effect of Pi depletion on *BNA* expression. However, this question is difficult to address directly. Given that Pi depletion decreases cellular ATP levels [39], while ATP depletion itself leads to NAD^+^ depletion [18], we would expect extended Pi-depletion to cause a corresponding drop in NAD^+^ levels. This would have the ultimate indirect consequence of limiting NAD^+^-dependent Hst1 activity and inducing *BNA* expression (Figure 2A), as Hst1 is a critical negative regulator of *BNA* expression [19,29]. Owing to these considerations, we attempted to identify a time window that would preserve the cellular NAD^+^ pool while also sufficiently inducing *PHO* signaling. We found that, soon after transfer to Pi-depleted (low-Pi) medium, cellular NAD^+^ levels significantly dropped (Figure 2B). Interestingly, one of the first evident changes upon Pi depletion was a redox imbalance between NADH/NAD^+^ (~5 min), with the ratio quickly rising and then leveling out over time. Since NAD^+^ depletion appears to be an inevitable confounding factor of Pi limitation, we employed the *hst1*Δ mutant in our studies of Pi depletion, which is not expected to be sensitive to changes in NAD^+^ levels with respect to the regulation of *BNA* expression. As shown in Figure 2D, the expression of most *BNA* genes was slightly but significantly reduced by Pi-depletion in *hst1*Δ cells, with the exception of *BNA6*. Expression of *PHO5* was included as a positive control of *PHO* activation. It is possible that *BNA6* expression is less sensitive to Pi depletion and that this effect is only visible under more derepressed conditions. To further investigate whether *BNA6* expression is indeed insensitive to Pi depletion, we sought to further increase *BNA* expression. It has been shown that the *ade16*Δ*ade17*Δ mutants, which are genetic mimics of adenine depletion, also have increased *BNA* expression through a mechanism in parallel to Hst1 (Figure 2C, left) [18]. In these cells, activation of *BNA* expression is mediated by a specific transcription complex Bas1–Pho2, which is stimulated by an adenine intermediate (5′-phosphoribosyl-5-amino-4-imidazole carboxamide monophosphate, ZMP) that it accumulates [18]. Although the primary function of Bas1–Pho2 complex is to activate genes for de novo adenine synthesis, it also activates *BNA* genes during adenine depletion or when cells accumulate ZMP metabolites [18]. Therefore, we also deleted *ADE16* and *ADE17* in the *hst1*Δ background in order to further induce *BNA* expression by this ZMP-Bas1–Pho2-dependent mechanism. Interestingly, expression of *BNA2* and *BNA6* was further increased in the *hst1*Δ*ade16*Δ*ade17*Δ triple mutant compared to the *hst1*Δ mutant (Figure 2D). In addition, in this background, we were able to observe that all *BNA* genes are sensitive to Pi-limitation, which reduces *BNA* gene expression and a concomitant increase in *PHO5* expression. These results also demonstrate an inverse correlation between *BNA* and *PHO* gene expression during Pi-limitation (Figure 2D).

Next, we examined whether *PHO* transcription activators might play a role in this co-regulation. Activation of *PHO* downstream genes is mediated by the Pho2–Pho4 transcription complex [31,32]. Interestingly, Pho2 also functions in the Bas1–Pho2 complex, which has been shown to be required for the activation of *BNA* genes in the adenine depletion mimicking *ade16*Δ*ade17*Δ mutants [18]. Although it is unclear whether Bas1–Pho2 has an effect on *BNA* expression in the WT background, these results suggest that Pho2 may be a limiting factor for the regulation of *BNA* and *PHO* gene expression. Supporting this, competition between the two complexes has been suggested for the regulation of *PHO* genes and adenine biosynthesis *ADE* genes [33]. To test this model, we first asked whether deleting *BAS1* would be sufficient to increase *PHO* expression and/or decrease *BNA* expression in the WT background without Pi-limitation. We found that *BAS1* deletion had no significant effect on the expression of *BNA*, *PHO5*, or *PHO8* in the WT background except for *BNA6* (Figure 2E). This suggests that the Bas1–Pho2 complex may only have a significant role as an activator of *BNA* expression under inducing conditions, for example, in the *ade16*Δ*ade17*Δ background [33]. It is also likely that each *BNA* gene is regulated differently under different conditions. However, it should also be considered that deletion of *BAS1* causes a significant reduction in cellular ATP levels [40,41], which would be likely to reduce NAD^+^ production downstream [18]. Therefore, *BNA* expression in *bas1*Δ cells may be confounded by reduction or partial loss of Hst1 activity, masking the potential effect of Bas1 itself, which might only become evident in the *hst1*Δ background. In this regard, we further examined the effect of *BAS1* deletion in *hst1*Δ cells, in which all *BNA* genes are de-repressed. As shown in Figure 2E, *BAS1* deletion significantly reduced the expression of most *BNA* genes in *hst1*Δ cells, confirming the role of Bas1 as a positive regulator of *BNA* expression, which acts independently of Hst1. Interestingly, unlike the other *BNA* genes, *BNA1* expression appears to be slightly increased in *bas1*Δ cells (Figure 2E), suggesting that the *BNA1* gene may be regulated by additional unique mechanisms distinct from those governing expression of the remaining set of *BNA* genes. Although it has been shown that Bas1–Pho2 is required for the activation of most *BNA* genes in *ade16*Δ*ade17*Δ cells [18,33], the effect of *bas1*Δ on *BNA* expression in a WT background has not been shown. Moreover, *hst1*Δ*bas1*Δ cells also showed a notable increase in *PHO5* and *PHO8* expression (Figure 2E), lending support to the model that Bas1 and Pho4 are in competition for limiting reserves of Pho2 (Figure 2C), and that this becomes visible in the absence of Hst1, the major regulator of *BNA* expression alongside Rpd3 [30]. These studies also firmly indicate that Bas1–Pho2 is required for optimal *BNA* expression in *hst1*Δ cells even under adenine-replete conditions.

Having observed a genetic interaction between the *hst1*Δ mutant and the *ade16*Δ*ade17*Δ (Figure 2D) and *bas1*Δ mutants (Figure 2E), we then examined whether Rpd3 also played a role in this context. Since Rpd3 antagonizes Hst1-mediated repression and promotes *BNA* expression, we examined whether Rpd3 was required for Bas1–Pho2-mediated activation of *BNA* expression in *ade16*Δ*ade17*Δ cells. As shown in Figure 2F, when we compared the *ade16*Δ*ade17*Δ and *rpd3*Δ*ade16*Δ*ade17*Δ mutants, *RPD3* deletion significantly reduced *BNA* expression in *ade16*Δ*ade17*Δ cells. Rpd3 may allow for improved recruitment of Bas1–Pho2 by preventing the spreading of repressive chromatin structure established by Hst1 [30]. However, the *rpd3*Δ*ade16*Δ*ade17*Δ mutant still expressed most *BNA* genes to a level higher than that of *rpd3*Δ cells, suggesting that the direct mechanism of Bas1–Pho2 recruitment might function independently of Rpd3.

### 2.3. The ade16Δade17Δ Mutant Exhibits Altered Cellular NR and NA-NAM Metabolism

**Figure 3:**
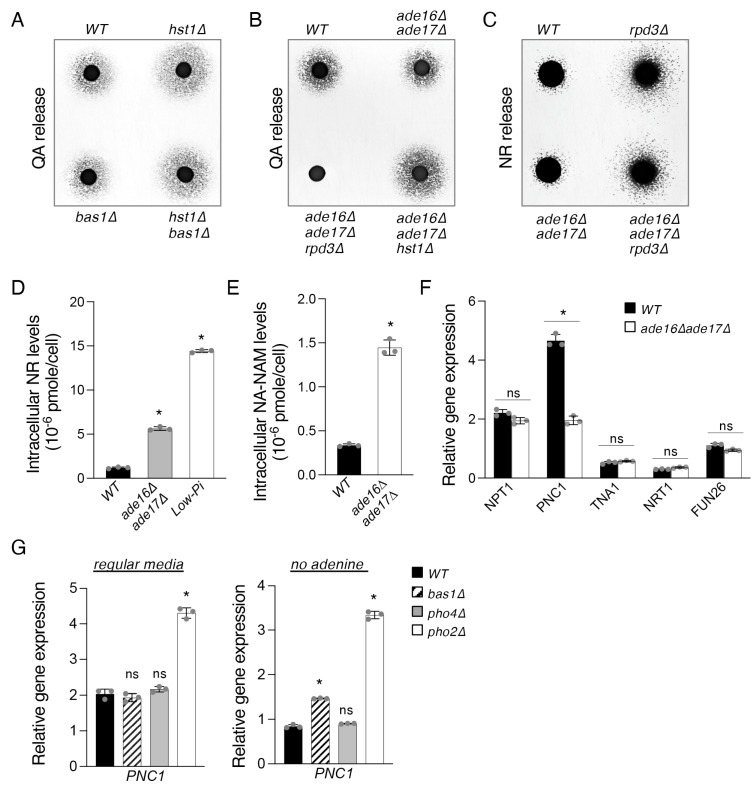
The *ade16*Δ*ade17*Δ mutant cells show altered homeostasis of NAD^+^ precursors. (**A**) Deletion of *BAS1* does not markedly affect QA release in WT or *hst1*Δ backgrounds. (**B**) Deletions of *ADE16* and *ADE17* decrease QA release in WT cells but do not visibly affect QA release in *rpd3*Δ or *hst1*Δ cells. (**C**) Deletions of *ADE16* and *ADE17* do not affect levels of NR release in WT and *hst1*Δ backgrounds. Feeder cell spots along with NR-dependent recipient cells (*npt1*Δ*bna6*Δ*pho5*Δ) were grown at 30 °C on a YPD plate for 3 days. (**D**) *ade16*Δ*ade17*Δ cells have higher intracellular NR levels relative to WT cells. WT cells transferred to low Pi for 30 min also produce more NR. (**E**) *ade16*Δ*ade17*Δ cells have higher intracellular NA-NAM levels relative to WT cells. (**F**) Expression of *PNC1*, responsible for the conversion of NAM to NA, is reduced in *ade16*Δ*ade17*Δ cells. *ade16*Δ*ade17*Δ cells do not show significant changes in expression of the NA/QA transporter Tna1 or the NR transporters Nrt1 and Fun26. Values shown are relative expression levels normalized to *TAF10* as a control. (**G**) Deletion of *PHO2* significantly increases expression of *PNC1* in both standard medium (left) and medium lacking adenine (right). Deletion of *BAS1* slightly increases *PNC1* expression in the medium without adenine. For (**D**–**G**), the graphs are representative of the trend observed across three independent experiments. Error bars represent data from three technical replicates for each strain in an experiment. The *p* values are calculated using Student’s *t*-test (*, *p* < 0.05; ns, not significant).

Having observed significant decreases in *BNA* expression when *BAS1* is deleted in the *hst1*Δ background, we sought to investigate what effect the loss of Bas1 might have on the production of QA, a de novo intermediate. Interestingly, *BAS1* deletion did not appear to decrease QA levels in either *WT* or *hst1*Δ cells (Figure 3A). It is likely that in *bas1*Δ cells, a small decrease in QA production may be masked by its decreased *BNA6* expression (Figure 2E), which is expected to cause QA accumulation (Figure 1A).

Surprisingly, although the *ade16*Δ*ade17*Δ mutant showed elevated *BNA* expression, these cells appeared to release less QA (Figure 3B). It is possible that a portion of produced QA is assimilated into NAD^+^ due to especially high *BNA6* expression in these cells (Figure 2E). This is likely the case since the *ade16*Δ*ade17*Δ mutant was shown to have increased levels of *BNA* expression and de novo pathway metabolites, as well as increased NAD^+^ levels in a medium lacking NA [18]. The QA production studies also further support Rpd3 as a primary positive regulator of *BNA* expression [30]. As shown in Figure 3B, the *rpd3*Δ*ade16*Δ*ade17*Δ mutant did not release detectable QA and displayed a similar phenotype observed in the *rpd3*Δ mutant (Figure 2A). In addition, unlike other *BNA* genes, *BNA1* expression was not increased in *ade16*Δ*ade17*Δ cells (Figure 2F), which may also contribute to observed lower QA levels (Figure 3B). These results also suggest some *BNA* genes may be independently regulated by different mechanisms. Next, we further examined whether other branches of NAD^+^ metabolism are also affected in the *ade16*Δ*ade17*Δ mutant. Since *PHO* signaling is activated in this mutant, we examined whether it also produced more NR, as observed in *rpd3*Δ cells. We found that although the *ade16*Δ*ade17*Δ mutant did not appear to release more NR (Figure 3C), its intracellular NR was significantly increased (Figure 3D). For comparison, direct Pi-depletion caused greater NR production in WT cells compared to the *ade16*Δ*ade17*Δ mutants (Figure 3D). Interestingly, the *ade16*Δ*ade17*Δ cells also showed a significant increase in intracellular NA-NAM levels (Figure 3E). This could be due to increased NR flow into the NA-NAM salvage pathway combined with a blockage in NA-NAM metabolism. To test this, we examined the expression of genes involved in NA (*NPT1*) and NAM (*PNC1*) metabolism. Moreover, higher intracellular NR and NA-NAM could also be due to increased import of these precursors, especially considering that the quantities of these metabolites released extracellularly are not visibly increased. Therefore, the expression of *TNA1* (NA and QA transporter), *NRT1* (NR transporter), and *FUN26* (vacuolar NR transporter) was also studied. As shown in Figure 3F, expression of *PNC1* was significantly reduced in *ade16*Δ*ade17*Δ cells, while expression of the other genes was not significantly altered. Interestingly, the observed inhibitory effect of *ade16*Δ*ade17*Δ on *PNC1* expression suggests that either the Bas1–Pho2 complex or the Pho2–Pho4 complex might negatively regulate *PNC1* expression, opposite the previously characterized roles of these complexes as activators of *ADE*/*BNA* expression [18,33] and *PHO* expression [42,43,44], respectively.

To test whether either of the two complexes regulates *PNC1* expression, we examined levels of *PNC1* expression in cells lacking Bas1, Pho4, and Pho2, respectively. We found that, under standard conditions, deletion of the shared subunit *PHO2* had the expected effect of significantly increasing *PNC1* expression, while deletion of the unique subunits *BAS1* and *PHO4* did not have a discernible effect on *PNC1* mRNA levels (Figure 3G, left panel). Next, we measured *PNC1* gene expression in a medium lacking adenine, a growth condition previously shown to promote the formation of the Bas1–Pho2 and Pho2–Pho4 complexes [33], expecting this to amplify any effect either complex might have. Under these conditions, we observed *PHO2* deletion to again cause a large increase in *PNC1* expression (Figure 3G, right panel). In addition, we found that in this case, deletion of *BAS1* caused a minor increase in *PNC1* expression, though far below the level shown in cells lacking the shared complex subunit Pho2. This suggests that both complexes may have overlapping roles in negatively regulating *PNC1* and thereby reducing NA-NAM salvage activity and that one complex is largely able to complement the role of the other in its absence. This establishes a novel role for Pho2 as a negative regulator of NA-NAM salvage, alongside its previously characterized roles as a positive regulator of both de novo NAD^+^ metabolism [18] and NR salvage [16]. Overall, our results showed that both increased NR production and a blockage in the conversion of NAM to NA (reduced *PNC1* expression) were observed in *ade16*Δ*ade17*Δ cells. Increased *PHO* signaling activity likely contributes to NR production [16], while reduced Pnc1 activity leads to the accumulation of NAM and NR. These results also suggest that increased *BNA* and *PHO* gene expression observed in the *ade16*Δ*and17*Δ mutants [33] may be partly due to NAM accumulation and reduced Hst1 activity. It has been shown that NAM can inhibit Hst1-activity [45,46] and de-repress *BNA* gene expression [19].

### 2.4. Pho84-Mediated Phosphate Transport Affects Homeostasis of NAD^+^ Precursors

**Figure 4:**
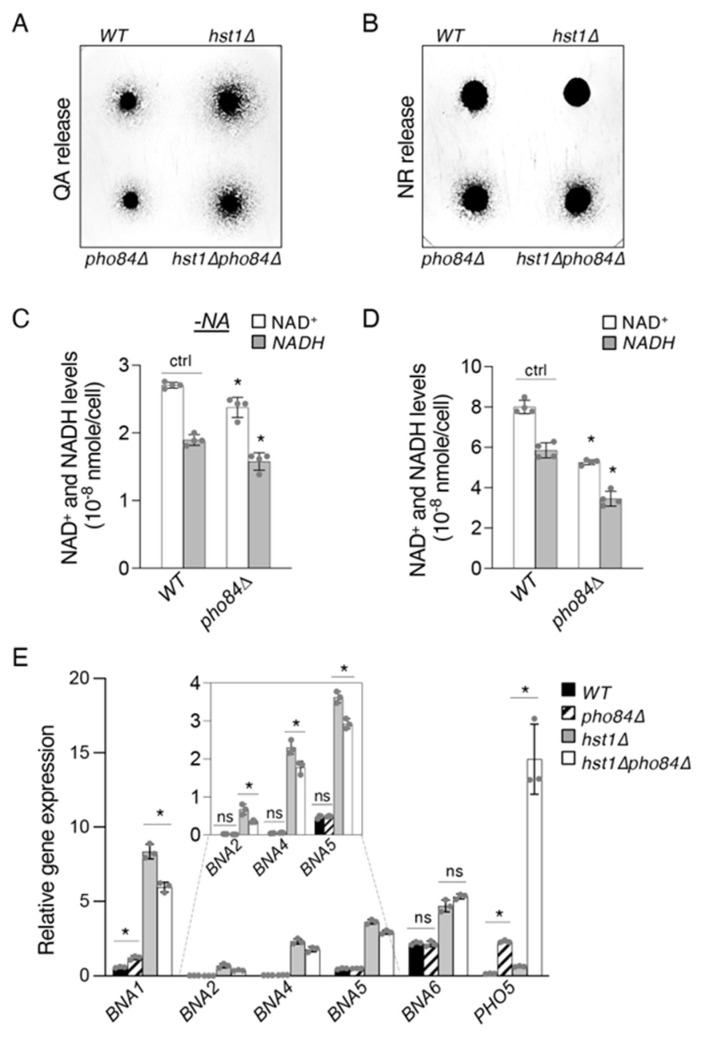
The phosphate transporter Pho84 influences cellular NAD^+^ production. (**A**) Deletion of *PHO84* slightly decreases QA release in WT and *hst1*Δ cells. (**B**) Deletion of *PHO84* slightly increases NR release in WT and *hst1*Δ cells. (**C**) Deletion of *PHO84* slightly reduces NAD^+^/NADH levels when cells are grown in SC medium lacking NA. (**D**) *pho84*Δ cells show a significant reduction of NAD^+^/NADH relative to WT cells in regular SC medium. (**E**) *pho84*Δ cells do not show markedly altered *BNA* expression compared to WT cells. *hst1*Δ*pho84*Δ cells exhibit small but significant reductions of *BNA* expression relative to *hst1*Δ cells. *PHO5* is included as a positive control for *PHO* activation. Values shown are relative expression levels normalized to *TAF10* as a control. For (**C**–**E**), the graphs are representative of the trend observed across three independent experiments. Error bars represent data from two biological replicates each with two technical replicates (for (**C**,**D**)) or from three technical replicates (for (**E**)) for each strain in an experiment. The *p* values are calculated using Student’s *t*-test (*, *p* < 0.05; ns, not significant; ctrl, control).

To further understand the connection between *PHO* activation and de novo NAD^+^ metabolism we employed the *pho84*Δ mutant as a genetic mimic of Pi depletion. Deletion of *PHO84* causes reduced intake and accumulation of Pi [44,47], which serves as a model of chronic reduction of Pi levels, as opposed to acute depletion resulting from short periods of growth in a low-Pi medium. First, we determined the effect of *PHO84* deletion on QA production in WT and *hst1*Δ cells. We found that the deletion of *PHO84* slightly, but visibly, reduces QA release in WT and *hst1*Δ backgrounds (Figure 4A). This may partly be due to competition between Bas1–Pho2 and Pho2–Pho4 caused by *PHO* activation, limiting the amount of Pho2 available for *BNA* induction. We also compared the levels of NR production in these cells and found that both *pho84*Δ and *hst1*Δ*pho84*Δ cells showed increased NR release relative to WT and *hst1*Δ cells, respectively. This suggests that Pi sensing, *PHO* activation, and NR metabolism remain largely functional in *hst1*Δ cells. Interestingly, *hst1*Δ*pho84*Δ cells appeared to release less NR compared to *pho84*Δ cells, suggesting that increased *BNA* expression might compete with *PHO* activation in the *pho84*Δ background.

Next, we examined whether the NAD^+^ pool is depleted by *PHO84* deletion. NAD^+^ levels were determined in cells grown in an SC medium lacking NA (in which NAD^+^ is derived mostly from de novo activity) (Figure 4C) and standard SC medium (Figure 4D). We found that NAD^+^ levels are slightly but significantly reduced in an SC–NA medium, supporting the hypothesis that *PHO* activation is able to reduce de novo activity. Consistent with the low Pi treatment results (Figure 2B), NAD^+^ levels were significantly reduced in *pho84*Δ cells grown in regular SC. This may be due to a limitation of Pi and, consequently, of the NA/NAM salvage pathway, which requires more ATP [18]. Lastly, the use of the *pho84*Δ mutant also allows us to further solidify the competitive model elaborated above (Figure 2C). We anticipate the competition to be more significant in the *hst1*Δ background in which *BNA* expression is induced and that reduced NAD^+^ levels caused by *pho84*Δ will not interfere with the analysis. Consistent with this expectation, we saw that expression of most *BNA* genes was slightly but consistently reduced in *hst1*Δ*pho84*Δ cells compared to *hst1*Δ cells (Figure 4E), closely resembling the differences observed by Pi depletion from the growth medium (Figure 2D).

### 2.5. Phosphate Depletion Increases Pho2 Binding Activity at the PHO5 Promoter but Not at the BNA2 Promoter

**Figure 5:**
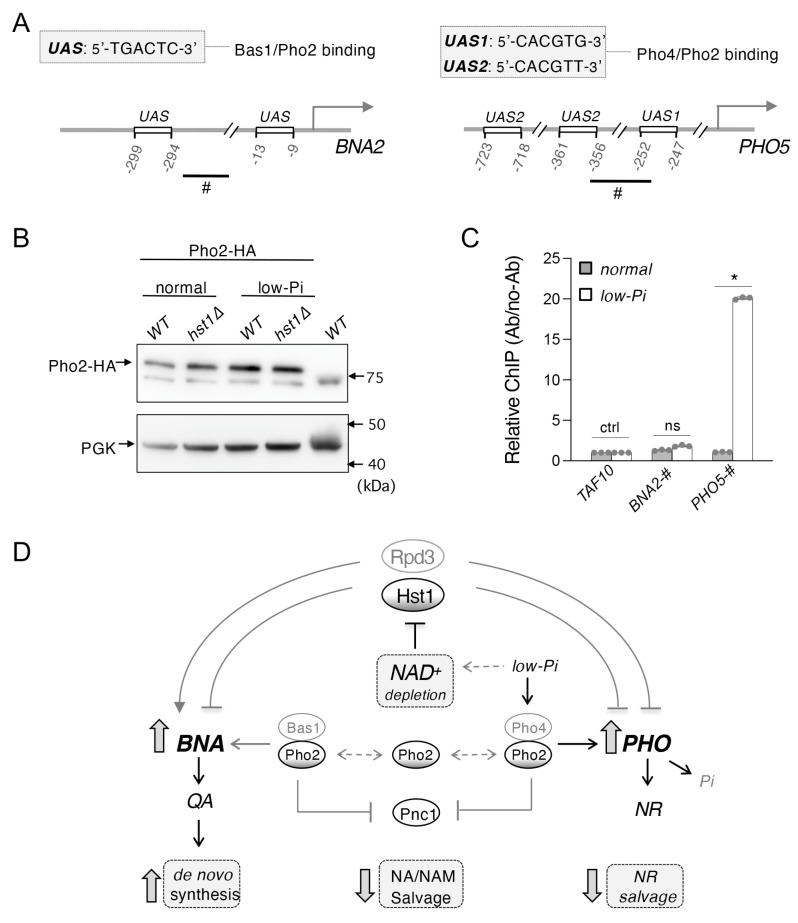
Model of the interconnection of NAD^+^ metabolism and *PHO* signaling by Hst1, Rpd3, and Pho2. (**A**) Schematics of putative Bas1 binding sites at the *BNA2* promoter (left) and of Pho4 binding sites at the *PHO5* promoter (right). Regions marked with ‘#’ were assayed for Pho2-HA binding. (**B**) Western blot analysis of Pho2-HA protein expression. Neither deletion of *HST1* nor 1 h of Pi depletion significantly affects levels of Pho2-HA expression relative to the PGK control. (**C**) Pho2-HA binding activities at *BNA2* and *PHO5* promoters determined by ChIP. Pi depletion is able to increase Pho2-HA binding to the *PHO5* promoter in *hst1*Δ cells. At the *BNA2* promoter, Pho2-HA appears to have low binding activities and Pi depletion does not further decrease its binding. Values shown are relative binding levels (Ab/no-Ab) normalized to *TAF10* as a control. Graphs are representative of the trend observed across three independent experiments. Error bars represent data from three technical replicates for each strain in an experiment. The *p* values are calculated using Student’s *t*-test (*, *p* < 0.05; ns, not significant). (**D**) Rpd3 is a positive regulator of de novo NAD^+^ metabolism, while Hst1 is a negative regulator. On the other hand, both appear to be negative regulators of the *PHO* pathway. Hst1 activity is decreased by low NAD^+^ levels, which may be caused by lowered ATP levels (not shown in this figure), which itself can be a consequence of phosphate depletion (low-Pi). This makes Hst1 sensitive to all three of these conditions, which consequently causes reduced silencing of *BNA* and *PHO* genes, leading to increased flux through de novo NAD^+^ biosynthesis and NR salvage pathways. Low-Pi may also enhance the formation of Bas1–Pho2 and Pho2–Pho4 complexes independent of NAD^+^ and Hst1, possibly via ZMP accumulation. Finally, low-Pi also promotes the translocation of Pho4 to the nucleus and increases Pho2–Pho4 complex formation. Under low-Pi conditions, the resulting lowered ATP and increased ZMP levels are predicted to activate Bas1–Pho2 and Pho2–Pho4, while low-Pi itself leads to prioritization of *PHO* activation via Pho4–Pho2 over *BNA* activation via Bas1–Pho2. Bas1–Pho2 and Pho2–Pho4 also inhibit NA-NAM salvage via negative regulation of *PNC1* expression. With its HDAC activity being independent of NAD^+^ levels, Rpd3 remains an activator of de novo NAD^+^ biosynthesis and NAD^+^ precursor transport, as well as a repressor of Pi sensing under all conditions described. Dashed lines indicate the mechanisms of these steps remain unclear. The *p* values are calculated using Student’s *t*-test (*, *p* < 0.05; ns, not significant; ctrl, control).

Lastly, we attempted to directly test the hypothesis that Pi depletion affects *BNA* expression via alteration of Bas1–Pho2 complex activity. Presumably, this would occur by increasing Pho2–Pho4 complex formation. It is possible that this increased formation of Pho2–Pho4 might reduce Bas1–Pho2 complex formation and limit the expression of the *BNA* genes. We again made use of the *hst1*Δ background in order to avoid the confounding influence of NAD^+^ limitation during the Pi depletion described above. To investigate this question, we focused on *BNA2* as the rate-limiting element of the de novo pathway which is consistently sensitive to factors associated with the general alteration of *BNA* expression [19]. The *BNA2* promoter region contains two predicted Bas1 binding sites near the transcription start site (Figure 5A, left); therefore, we assessed Pho2 binding in the short region between these sites using chromatin immunoprecipitation (ChIP) of Pho2 tagged with an HA epitope. In addition, we investigated binding to the *PHO5* promoter as a positive control for *PHO* induction. The binding sites of Pho4 and Pho2 at the *PHO5* promoter are well characterized [48,49,50], so we measured Pho2 binding in a region previously associated with Pho4/Pho2 binding, directly upstream of the *UAS1* binding site (Figure 5A, right). First, we examined the expression of the Pho2-HA protein under the conditions investigated by Western blotting. As shown in Figure 5B, Pho2-HA expression was not visibly altered relative to the PGK control either by deletion of *HST1* or by depletion of Pi.

As expected, Pi depletion caused a significant increase in Pho2 binding to the *PHO5* promoter, confirming the successful operation of *PHO* signaling under the conditions investigated (Figure 5C). However, Pi depletion did not appear to significantly alter Pho2 binding to the *BNA2* promoter. Of note is that an overall low Pho2 binding activity was observed at the *BNA2* promoter, making it difficult to detect a further reduction in Pho2 binding by a low Pi. It is possible that Pho2 only transiently associates with the *BNA2* promoter and that our assay conditions are not optimal to capture Pho2 binding. The mechanism of Bas1–Pho2 activity at the *BNA2* promoter remains largely unknown; for instance, whether one of the two proteins is responsible for the recruitment of the other, or whether the putative binding sites in Figure 5A are genuine binding sites for Bas1–Pho2. It remains to investigate this complex issue in greater detail in future studies.

## 3. Discussion

In this study, we show that Hst1 and Rpd3 play a role in co-regulating Pi-sensing *PHO* signaling and de novo NAD^+^ metabolism (Figure 5D). Together with their antagonistic regulation of de novo metabolism [30], the two appear to employ a different mode of regulation at the *PHO5* and *PHO8* promoters, with both serving as negative regulators in this latter case (Figure 1B). Two transcription complexes, Pho2–Pho4 and Bas1–Pho2, are also active in this connection. The Pho2–Pho4 complex has long been known as an activator of *PHO* pathway targets [42,43,44], while the Bas1–Pho2 complex was recently identified as a positive regulator of *BNA* expression in the *ade16*Δ*ade17*Δ background [18] (Figure 5D). The sharing of Pho2 between these complexes provides an opportunity for coordination between de novo NAD^+^ synthesis and *PHO* signaling pathways, considering that Pi depletion is also associated with the reduction of cellular NAD^+^ levels (Figure 2B). To study the specific effect of Pi-depletion on *BNA* expression independent of NAD^+^ levels, we employed the *hst1*Δ mutant and showed that Pi-depletion in fact limits *BNA* expression in *hst1*Δ cells (Figure 2D). These results indicate some degree of competition between Bas1 and Pho4 for binding to Pho2, which has also been suggested to occur between the *ADE* and *PHO* genes [33]. The ultimate consequence of this would be two competing signals induced by low-Pi: low NAD^+^ promotes *BNA* activation via loss of Hst1 activity, while *PHO* activation also limits *BNA* expression (Figure 5D). This effect indeed becomes even more pronounced in *hst1*Δ*ade16*Δ*ade17*Δ cells. Supporting this model, we showed that deleting *BAS1* indeed reduces *BNA* expression with a concomitant increase in *PHO* gene expression in *hst1*Δ cells (Figure 2E). On the other hand, the deletion of *BAS1* alone does not significantly alter *BNA* expression. It appears that the Bas1–Pho2 complex is not a major regulator of *BNA* expression under standard conditions, but that it is necessary for full *BNA* induction under inducing conditions, such as in *hst1*Δ or *ade16*Δ*ade17*Δ cells. As noted previously, however, the low ATP levels seen in *bas1*Δ cells [40,41] may cause declines in NAD^+^ production and loss of Hst1 activity, confounding straightforward investigation of *BAS1* deletion in the WT background.

As a sirtuin, Hst1 activity is limited by low NAD^+^, resulting in the de-repression of both the *BNA* genes and the *PHO* pathway (Figure 5D). Owing to these relationships, *PHO* gene expression is predicted to be activated in low-Pi conditions not only by the main mechanism of Pho4 translocation to the nucleus but also indirectly by the reduction of NAD^+^ levels under low-Pi conditions (Figure 2A). This series of events ultimately serves to strongly activate *PHO* targets, one result of which is the increased production of NR (Figure 3D and Figure 5D). We have also identified novel and distinctive roles for Bas1–Pho2 and Pho2–Pho4 as negative regulators of NA-NAM salvage via *PNC1*. The *ade16*Δ*ade17*Δ mutant cells show reduced *PNC1* expression (Figure 3F) and, consequently, NAM accumulation (Figure 3E) (Pnc1 converts NAM to NA). In a medium without adenine, cells lacking Pho2, and to a markedly lesser degree Bas1, exhibit increased *PNC1* expression. The mode of *PNC1* regulation we have observed greatly helps to contextualize and clarify the nexus of Pi, ATP, and NAD^+^ metabolism described here. It was shown previously that making NAD^+^ from NA-NAM salvage requires more ATP than from the de novo pathway, due to additional ATP consumption by Npt1, directly downstream from Pnc1 [18]. Therefore, reduced *PNC1* expression may help decrease NA-NAM salvage activity to preserve ATP for other cellular functions and alleviate NA accumulation behind Npt1. Increased NAM accumulated behind Pnc1 may also help de-repress *BNA* and *PHO* genes to facilitate de novo NAD^+^ synthesis and *PHO* activation in a Hst1-dependent manner, as NAM is a strong inhibitor of Hst1 [45,46]. At the same time, Bas1–Pho2 promotes *BNA* expression and helps to favor the usage of de novo NAD^+^ metabolism over NA-NAM salvage. Of note, low Pi directly leads to increased Pho2–Pho4 activity and also low ATP levels, thereby also indirectly promoting Pho2–Pho4 and Bas1–Pho2 activity. Taken together, this suggests that low Pi causes redirection of cellular NAD^+^ metabolism from NA-NAM salvage to de novo biosynthesis through the following set of events: promotion of *BNA* expression via Bas1–Pho2 as well as reduced Hst1 activity, reduction of Npt1 catalytic activity via limitation of ATP [18], and, finally, reduction of Pnc1 activity via the combined effect of Pho2–Pho4 and Bas1–Pho2 (Figure 5E). Lastly, the apparent competition between Bas1 and Pho4 for Pho2 under low-Pi conditions seems to favor *PHO* activation (Figure 2D and Figure 4E) and may help to set an upper limit to *BNA* activation under these conditions.

On the other hand, Rpd3 serves as an epigenetic regulator in this metabolic network independent of NAD^+^ levels. Quite interestingly, Rpd3 has a remarkably high degree of overlap with each of the regulators described above: it is a positive regulator of the *BNA* genes, *PNC1* [30], and a negative regulator of the NR-producing phosphatases Pho5 and Pho8 [34,35] (Figure 1C). This helps ensure that *PHO* activity, de novo NAD^+^ metabolism, as well as salvage of NA, NAM, and NR remain controlled to some degree, even when levels of NAD^+^ and Pi are low. For example, in the case of *PHO5* expression and NR production, the removal of both Hst1 and Rpd3 leads to synergistically increased activity, which may be detrimental to cells. However, Rpd3 is required not only as a *PHO* repressor but also seems to be required for full induction of *PHO5* [34,51], highlighting the intricate role of Rpd3 as both a positive and negative epigenetic regulator depending on the context. As noted above, Rpd3 acts as a positive regulator of the *BNA* genes, as well as of the NR transporter *NRT1* and the NA/QA transporter *TNA1*, all of which are induced in *hst1*Δ or by limitation of NAD^+^ [19]. Interestingly, Nrt1 has also been shown to be a transporter of the ZMP precursor 5-Aminoimidazole-4-carboxamide-1-β-d-ribofuranoside (AICAR) [52]. Collectively, these results provide a model connecting de novo metabolism, *PHO* signaling, as well as salvage of NA, NAM, and NR.

As anticipated from the preceding relationship, the phosphate transporter Pho84 also appears to have a significant influence on the regulation of de novo NAD^+^ metabolism. It is likely that this primarily proceeds through the model outlined, as loss of *PHO84* is associated with reduced accumulation of Pi [47] and high levels of *PHO* induction (Figure 4E) [53]. As mentioned above, the relationship is likely twofold: loss of Hst1 activity due to decreased NAD^+^ production and decreased availability of Pho2 via its increased recruitment into the *PHO*-activating Pho2–Pho4 complex. These two influences are predicted to have opposite effects on *BNA* expression, and the effect of reduced Hst1 activity would be predicted to predominate based on its significant control of *BNA* expression [19,29,30] relative to the more minor effect exerted by Bas1–Pho2 [18] (Figure 2E and Figure 3A). However, *pho84*Δ cells show almost no changes in *BNA* expression relative to WT cells (Figure 4E), suggesting that the two influences may be more balanced in degree. This could possibly be due to an incomplete limitation of Hst1 activity at intermediate intracellular NAD^+^ concentrations, or alternatively due to particular adaptations to chronically low Pi in *pho84*Δ cells (Figure 4D).

In addition, the effect of Rpd3 on the transport of Pi via Pho84 cycling is a topic in need of further investigation. Having established the close relationship of both Pi-sensing and Rpd3 with the regulation of de novo metabolism, the two may interact in a variety of ways to influence the ultimate status of *BNA* gene expression. For instance, *rpd3*Δ cells are known to accumulate less Pi due to defective recycling of Pho84, causing a significant *PHO* induction independently of Rpd3 activity at *PHO* promoters [35]. This suggests that Pho4 may bind and sequester some Pho2 away from Bas1, thereby indirectly reducing *BNA* expression. Although this effect is expected to be minor in comparison with the effect of Rpd3 as an epigenetic regulator of *BNA* expression per se, it provides a hint of the complexity involved in joining de novo NAD^+^ metabolism, Pi-sensing, and NR salvage. Moreover, Pho84 recycling defects in *rpd3*Δ cells may partially explain the low NAD^+^ phenotype of *rpd3*Δ cells in standard and -NA media [30].

Finally, under our experimental conditions, we did not observe a direct limitation of Pho2 binding to the *BNA2* promoter after Pi depletion that would directly confirm the model of competition between Bas1–Pho2 and Pho2–Pho4 described above. However, neither does this observation unambiguously exclude the possibility of competition between the two complexes for limiting reserves of Pho2. Several considerations remain to be addressed in this respect. Namely, the promoter of *BNA6* may provide a useful alternative to the *BNA2* promoter in capturing alterations of Bas1–Pho2 complex activity and binding; among all the *BNA* genes, *BNA6* expression seems to be the most sensitive to deletion of *BAS1* (Figure 2E). The *BNA6* promoter is also less characterized relative to that of *BNA2*; studies of *BNA6* and other *BNA* genes may therefore provide insights into different forms of regulation among the set of *BNA* genes and reveal elements that are unique to each. In addition, the mechanism of Bas1–Pho2 binding to the *BNA2* promoter is largely unexplored. It may be that any influence of Pho2 sequestration on *BNA* expression is not visible in a phenomenon as facile as the simple quantity of Pho2 binding. It was earlier demonstrated that Bas1 is responsible for recruiting Pho2 to the *ADE* promoters [54], but this may not necessarily be the case for the *BNA* promoters. It may indeed be the case that Pho2 binds to the *BNA* promoters first and later assists Bas1 binding, and that limitation of Pho2 availability is only visible as a decrease in Bas1 abundance at the *BNA2* promoter Alternatively, some unknown modification or factor downstream of Bas1–Pho2 might provide a better readout of this phenomenon. Finally, two opposite influences are rendered by Pi depletion. These are the increased formation of Pho2–Pho4 described above as well as the limitation of ATP [39], which is predicted to cause increased ZMP levels. Assuming that limiting Pho2 levels do indeed engender competition between Bas1 and Pho4, this influence may be somewhat offset by the promotion of Bas1–Pho2 complex formation by ZMP. This level of context-dependence and complexity makes the question a difficult one to resolve definitively.

In summary, Hst1, Rpd3, and Pho2 appear to integrate the regulation of several disparate branches of NAD^+^ metabolism, and their activities and targets also provide links to Pi sensing, purine metabolism, and the overall status of the cell’s NAD^+^ pool. This set of regulators, therefore, helps to coordinate a variety of interrelated metabolic signals in budding yeast. Further work will be required to explore the mechanistic interactions among these regulators and to establish the means by which they compete and cooperate to influence the cellular pools of NAD^+^ and its precursors. Altogether, this work contributes to the elaboration of the relations by which NAD^+^ metabolism is governed and helps to connect different branches of NAD^+^ metabolism among each other and with other signaling networks in the cell.

## 4. Materials and Methods

### 4.1. Yeast Strains, Growth Media, and Plasmids

The parental WT yeast strain BY4742 *MATα his3*Δ*1 leu2*Δ*0 lys2*Δ*0 ura3*Δ*0* used for this study was acquired from Open Biosystems [55]. Standard yeast growth media including synthetic minimal (SD), synthetic complete (SC), and yeast extract/peptone/dextrose (YPD) rich media were made as described [56]. Niacin-free yeast nitrogen base acquired from Sunrise Science Products was used to make special NA-free SD and NA-free SC. Low phosphate (low-Pi) medium was prepared by phosphate precipitation from SD or SC as previously described [16]. In brief, for each liter of low-Pi SC or SD, 2.46 g of MgSO_4_ were first dissolved in SC or SD. Next, 8 mL of concentrated ammonia was slowly added with gentle stirring to precipitate inorganic phosphate as MgNH_4_PO_4_. After filtration, HCl was added to the clear solution to adjust pH to 6, followed by autoclave sterilization. Additional auxotrophic supplements and glucose were added after autoclave. All gene deletions were done by replacing the coding regions of WT genes with gene-specific DNA fragments generated by PCR using either the p*AG32-hphMX4* [57] or the reusable *loxP*-*kanMX-loxP* (pUG6) [58] cassette as a template. Double gene deletions were carried out by employing different drug resistant markers for each round of gene deletion. Multiple gene deletions were achieved by using the galactose-inducible Cre recombinase to remove the *loxP*-*kanMX-loxP* marker from the yeast genome [58], followed by another round of gene deletion. The HA epitope tag was added to target genes directly in the genome using the pFA6a-3HA-kanMX6 or pFA6a-kanMX6-PGAL1-3HA plasmid as a template for PCR-mediated tagging [59].

### 4.2. Quantitative PCR (qPCR) Quantitation of Gene Expression

For each yeast strain, we collected approximately 40–50 OD_600_ unit (absorbance at 600 nm; 1 OD_600_ unit = ~1 × 10^7^ cells/mL) cells grown to early-logarithmic phase in SD (~6 h growth from OD_600_ of 0.1) by centrifugation. Collected cells were immediately subjected to RNA extraction. Total RNA was isolated using GeneJET RNA purification Kit (Thermo Scientific, Waltham, MA, USA) and cDNA was synthesized using QuantiTect Reverse Transcription kit (Qiagen, Hilden, Germany). About 50–100 ng of cDNA and 500 nM of each primer (final concentrations) were used for each 20 µL qPCR reaction. qPCR reactions were run in 96-well plates on Roche LightCycler 480 using LightCycler 480 SYBR green I Master Mix (Roche, Basel, Switzerland) as described [19]. The average size of the amplicon was ~120 bp for each gene of interest. The target mRNA transcript levels were normalized to *TAF10* transcript levels.

### 4.3. Repressible Acid Phosphatase (rAPase) Activity and Alkaline Phosphatase (rALPase) Assays

The *rAPase* liquid assay was modified from a protocol as previously described [60]. For each strain, about 2.5 OD_600_ unit cells grown in SC for 6 h were collected, washed, and resuspended in 150 μL sterile water resulting in cell suspension. Next, 600 μL of substrate solution (5.6 mg/mL *p*-nitrophenylphosphate, pNPP, in 0.1 M sodium acetate, pH 4) was added to the cell suspension, followed by incubation at 30 °C for 15 min. To stop the reaction, 600 µL ice-cold 10% trichloroacetic acid was added. Next, 600 μL of this final mixture was mixed with 600 μL saturated Na_2_CO_3_ to allow color (neon yellow) development. Cells were then removed by centrifugation to acquire the supernatant for OD_420_ colorimetric phosphatase product *p*-nitrophenol (pNP) determination. The *rAPase* activities were determined by normalizing OD_420_ readings to the total cell number (OD_600_). The cell extract-based *rALPase* activity assay was carried out as previously described with modifications [61]. In brief, about 2.5 OD_600_ units of cells grown in SC for 6 h were collected and washed in 0.85% NaCl with 1 mM PMSF by centrifugation [61]. The resultant cell pellet was then resuspended in 600 µL lysis buffer (20 mM PIPES, 0.5%, Triton X-100, 50 mM KCl, 100 mM potassium acetate, 10 mM MgSO_4_, 10 μM ZnSO_4_, 1 mM PMSF) followed by bead-beating. The resultant cell lysates were then centrifuged at 13,200 rpm for 5 min at 4 °C to collect the supernatant. Enzymatic reactions were carried out by mixing 200 µL supernatant with 300 µL reaction buffer for each sample (333 mM Tris-HCl, pH 8.5, 0.53% Triton X-100, 133 mM MgSO_4_, 13.3 µM ZnSO_4_, w/wo 1.66 mM pNPP), followed by incubation at 37 °C for 20 min. Reactions were stopped by adding 500 µL stop buffer (1 M glycine/KOH, pH 11.0). Supernatants were then collected by centrifugation, and the *rALPase* activities were determined by normalizing *A*_420_ readings of colorimetric phosphatase product pNP to the total cell number (OD_600_) used in each reaction.

### 4.4. QA and NR Cross-Feeding Plate Assays

These assays have been used as readout for relative QA and NR levels in previous studies [16,19,30]. Specific mutants, which depend on QA or NR for growth, were employed as the “recipient cells”, and yeast strains of interest were the “feeder cells”. Note that standard growth media do not contain the NAD^+^ intermediates such as NR and QA needed for the growth of recipient cells. Therefore, the extent of the recipient cell growth indicates the relative levels of QA and NR released by feeder cells. In brief, recipient cell suspension (made in sterile H_2_O) was plated onto a solid agar plate (~10^4^ cells/cm^2^). Next, ~2 × 10^4^ cells of each feeder cell strain (2 µL cell suspension made in sterile water at OD_600_ of 1) were spotted on the lawn of recipient cells. Plates were incubated at 30 °C for 2–3 days and then the images were captured. QA cross-feeding was carried out on SC or SD using the QA-dependent *npt1*Δ*nrk1*Δ*bna4*Δ mutant. NR cross-feeding was carried out on YPD or SC using the NR-dependent *npt1*Δ*bna6*Δ*pho5*Δ mutant.

### 4.5. Measurement(s) of NAD^+^, NADH, QA, NR, and NA-NAM

Total intracellular levels of NAD^+^ and NADH were determined using enzymatic cycling reactions as previously described [62] with modifications. Approximately 1 OD_600_ unit cells grown to early-logarithmic phase in SC (~6 h growth from OD_600_ of 0.1) were collected in duplicate by centrifugation. Acid extraction was performed in one tube to obtain NAD^+^, and alkali extraction was performed in the other to obtain NADH at 60 °C for 40 min. Amplification of NAD^+^ or NADH in the form of malate was carried out using 2–3 µL (for NAD^+^) or 4 µL (for NADH) of neutralized acid or alkali-extracted lysate in 100 µL of cycling reaction at room temperature for 1 h. Reactions were terminated by heating at 100 °C for 5 min. Malate produced from the cycling reaction was converted to oxaloacetate and then to aspartate and a-ketoglutarate. The reaction produced a corresponding amount of fluorometric NADH as readout, which was measured with excitation at 365 nm and emission monitored at 460 nm. Standard curves for determining NAD^+^ and NADH concentrations were obtained by adding NAD^+^ and NADH into the acid and alkali buffer (at final concentrations of 0, 2.5, and 7.5 µM) and then were treated with the same procedure along with other samples. Measurements of the intracellular and released levels of NAD^+^ intermediates (QA, NR, and NA-NAM) were determined by a liquid-based cross-feeding bioassay as previously described [16,37,63]. To prepare cell lysate for intracellular NAD^+^ intermediates determination, about 200 OD_600_ unit (for NR and NA-NAM) or 900 OD_600_ unit (for QA) cells of interest (donor cells) grown to late-logarithmic phase in SC (~16 h growth from an OD_600_ of 0.1) were collected by centrifugation and lysed by bead-beating (Biospec Products, Bartlesville, OK, USA) in 400 μL (per 200 OD_600_ unit cells) ice-cold 50 mM ammonium acetate solution. The supernatant was collected by centrifugation and the pellet was extracted two more times with 600 µL ice-cold 50 mM ammonium acetate solution, which resulted in ~1.6 mL cell lysate. After filter sterilization, 100–200 μL of clear extract (2.5 mL for QA) was supplemented to 8 mL of specific NAD^+^ intermediate-dependent recipient cells with a starting OD_600_ of 0.05 in SC. Supplemented cells were then grown at 30 °C for 24 h. For determining released extracellular NAD^+^ intermediates levels, 20 mL growth medium from the culture of cells of interest was collected and filter-sterilized, and then 4 mL was added to recipient cell culture in 2× SC to a final volume of 8 mL with a starting OD_600_ of 0.05. Control cultures of recipient cells in SC without supplementation were included in all experiments. For measuring relative QA levels, the QA-dependent *npt1*Δ*nrk1*Δ*bna4*Δ or *npt1*Δ*nrk1*Δ*bna1*Δ mutants were used as recipient cells. For measuring relative NR levels, the NR-dependent *npt1*Δ*bna6*Δ*pho5*Δ mutant was used as recipient cells. To measure relative NA-NAM levels, the NA-NAM dependent *bna6*Δ*nrk1*Δ*nrt1*Δ mutant was used as recipient cells, and the cultures were grown in NA-free SC. After incubation at 30 °C for 24 h, the growth of the recipient cells (OD_600_) was measured and normalized to the cell number of each donor strain used. Normalized OD_600_ readings were then converted to concentrations of QA, NR, and NA-NAM using the standard curves established as previously described [37,63].

### 4.6. Protein Extraction and Western Blot Analysis

Approximately 20 OD_600_ unit cells grown in SC to early-logarithmic phase (OD_600_ of ~1) were collected by centrifugation. Collected cells were snap-frozen in LN_2_ or immediately subjected to cell extraction. The cell lysate was obtained by bead-beating at 4 °C for 20 min in lysis buffer (50 mM Tris-HCl, pH 7.5, 100 mM NaCl, 1% Triton X-100, 5 mM EDTA, pH 8, 1 mM PMSF, and protease inhibitor cocktail, Pierce). Protein concentrations were measured using the Bradford assay (Bio-Rad, Hercules, CA, USA) and 20 µg (Pho2-HA) or 10 µg (PGK) of total protein was loaded in each lane. After electrophoresis, proteins were transferred to a methanol-activated polyvinylidene fluoride membrane (PVDF, GE Healthcare, Amersham, UK). Blocking was carried out using OneBlock™ Western-CL Blocking buffer at room temperature for 1 h. The membranes were then washed and blotted with either an anti-HA rabbit antibody (Cell Signaling 3724S, Danvers, MA, USA) or an anti-PGK mouse antibody (Invitrogen 459250, Waltham, MA, USA) at 4 °C overnight. Proteins were visualized using anti-mouse (for PGK) or anti-rabbit (for HA) IgG antibody conjugate to horseradish peroxidase (Invitrogen) and the ECL reagents (Amersham, GE). The chemiluminescent image was analyzed using the Amersham Imager 600 (GE, Amersham, UK) system and software following the manufacturer’s instructions.

### 4.7. Chromatin Immunoprecipitation (ChIP) Assay

Approximately 300 OD_600_ unit cells grown to early-logarithmic phase in SD for 6 h, as well as an equivalent number of cells grown for 5 h in SD and 1 h in SD without Pi, were crosslinked with 1% formaldehyde for 30 min at room temperature and stopped by adding glycine to a final concentration of 125 mM. Cells were pelleted by centrifugation and washed two times with cold Tris-buffered saline (20 mM Tris-HCl, pH 7.5, 150 mM NaCl). Cells were lysed by bead beating in 1 mL of lysis buffer (50 mM HEPES, 140 mM NaCl, 1% Triton X-100, 1 mM EDTA, 0.1% sodium deoxycholate, 0.1 mM PMSF, and protease inhibitor cocktail, Pierce) [19,30,64]. The cell lysate was drawn off the beads and centrifuged at a maximum speed (13,200 rpm) for 30 min at 4 °C. The chromatin pellet was resuspended in 1 mL of lysis buffer and sonicated on ice eight times with 20 s pulses using a Branson 450 Sonicator (Thomas Scientific, Swedesboro, NJ, USA) (output control set at 1.5 and duty cycle held at constant) to shear chromatin to an average length of ~500 bp. Sonicated chromatin solution was centrifuged twice at 10,000 rpm for 10 min at 4 °C. The supernatant was then aliquoted into two tubes (labeled “IP”, immunoprecipitated, and “no-Ab”, no antibody). The IP samples were incubated overnight at 4 °C with anti-HA monoclonal antibody (Abcam ab1424 or Cell Signaling 3724S) at a dilution of 1:150 (Abcam, Cambridge, UK) or 1:50 (Cell Signaling, Danvers, MA, USA). Both IP and no-Ab samples were incubated with 60 μL of ChIP-grade protein G beads (Cell Signaling 9007S) for 2 h at 4 °C and then washed as previously described. DNA was then eluted from the beads two times with 125 μL of elution buffer (5 × TE, 1% SDS). The combined DNA solution and input samples were incubated at 65 °C overnight to reverse the crosslinking. The purified DNA samples were analyzed by qPCR as described above. The amount of immunoprecipitated specific promoter DNA was determined relative to no-Ab DNA.

## Data Availability

All data are contained within this article.
